# The effectiveness of a body positioning device for controlling patient movement and additional sedative use during endoscopic retrograde cholangiopancreatography: A retrospective analysis

**DOI:** 10.1002/deo2.70095

**Published:** 2025-03-11

**Authors:** Haruka Masuda, Tsutomu Nishida, Kengo Matsumoto, Dai Nakamatsu, Shiro Hayashi, Masashi Yamamoto

**Affiliations:** ^1^ Department of Gastroenterology Toyonaka Municipal Hospital Osaka Japan; ^2^ Department of Gastroenterology and Internal Medicine Hayashi Clinic Osaka Japan

**Keywords:** body movement control, body positioning device, endoscopic retrograde cholangiopancreatography, sedation, sedation‐related complications

## Abstract

**Background:**

Endoscopic retrograde cholangiopancreatography requires precise body movement control for procedural safety and efficiency. Sedatives are commonly used but pose risks, especially in elderly patients. This study evaluated the effectiveness of the Medo V‐Fix device in controlling patient movement during endoscopic retrograde cholangiopancreatography.

**Methods:**

Of 1723 endoscopic retrograde cholangiopancreatography procedures performed between January 2021 and March 2024, 1,528 were analyzed after excluding cases with missing data. Patients were divided into two groups, the device group (*n* = 697) and the nondevice group (*n* = 831). The groups were compared with respect to body movement control, additional sedative administration, sedation‐related complications, and procedure discontinuation.

**Results:**

Baseline characteristics were similar between the groups. Body movement control was better with the device (good, 65.7%; poor, 24.0%; and very poor, 10.3%) than without it (good, 48.1%; poor, 30.7%; and very poor, 21.2%; *p* < 0.0001). The device reduced the need for manual assistance and additional sedatives. Fewer patients in the device group (9.5% vs. 15.6%, *p* = 0.0003) required an additional thiopental dose, and the dose was lower (4.5 mg vs. 6 mg, *p* = 0.0015). No procedure discontinuation occurred in the device group, whereas five discontinuations occurred in the nondevice group. Although hypoxemia was more frequent in the device group (14.5% vs. 8.8%, *p* = 0.0005), no severe adverse events occurred.

**Conclusions:**

The Medo V‐Fix device significantly improved body movement control and reduced the need for additional doses of sedatives and manual intervention. Despite a higher incidence of mild hypoxemia, these events were appropriately managed with routine monitoring, indicating that the device increases procedural safety and efficiency.

## INTRODUCTION

Endoscopic retrograde cholangiopancreatography (ERCP) is a technically demanding procedure that requires precise patient safety and efficiency.^1^ Uncontrolled patient movement during ERCP significantly compromises both of these requirements. To mitigate this risk, sedatives stabilize patients.^2^, ^3^ However, in elderly patients, sedatives increase the risk of sedation‐related complications (SRCs).^4^ Furthermore, guidelines recommend that an anesthesiologist supervises high‐risk patients, which may limit procedural flexibility.^3^ This additional requirement for an anesthesiologist in high‐risk cases may limit the use of alternative methods to effectively control patient movement.

Given these challenges, physical restraints are promising alternatives for controlling patient movement during ERCP. Restraints can restrict patient movement, thereby enhancing the safety and precision of the procedure. Despite the risk of complications, the benefits outweigh the risks, and patients should receive education and provide consent prior to undergoing the procedure. Studies suggest that physical restraints may reduce the need for sedatives and shorten recovery time,^5^ which is particularly beneficial for elderly patients.

Medo V‐Fix (Century Medical Inc.) is a body positioning device designed to improve patient stability that was introduced in November 2022. This user‐friendly device is similar to other devices, such as EZ‐FIX.^5^ While Medo V‐Fix utilizes beads and a compressor for adjustable decompression, EZ‐FIX employs polystyrene particles and vacuum sealing. This study retrospectively evaluated the effectiveness of the Medo V‐Fix device during ERCP, focusing on its impact on body movement control, patient safety, and the need for sedatives.

## METHODS

### Patients

This single‐center retrospective observational study analyzed ERCP procedures consecutively performed at Toyonaka Municipal Hospital between January 2021 and March 2024. Of the 1723 procedures, 1528 were included after excluding cases with incomplete data. The patients were divided into two groups: the device group (*n* = 697) and the nondevice group (*n* = 831). Owing to the limited availability of the device, some procedures were conducted without it, resulting in a mixed sample.

### Ethics

The study adhered to the principles of the Declaration of Helsinki and was approved by the Ethics Committee of Toyonaka Municipal Hospital (2024‐05‐02). The need for informed consent was waived through the opt‐out method on our hospital's website due to the retrospective nature of the study.

### Body positioning device

**FIGURE 1:**
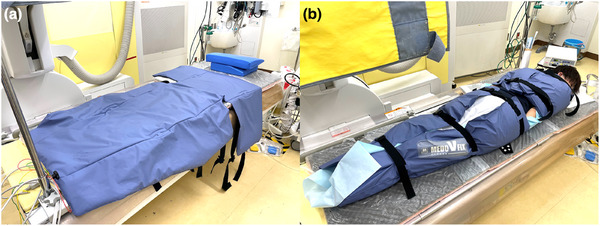
The Medo V‐Fix body positioning device. This specialized device secures patients during endoscopic procedures, ensuring stability and comfort (a). It allows for precise positioning and reduces movement, which can compromise procedural accuracy and safety. The Medo V‐Fix is adjustable to fit various patient sizes, ensuring optimal immobilization without causing discomfort (b). It is an essential tool for enhancing procedural efficiency and improving the overall quality of endoscopic examinations.

The Medo V‐Fix device (Century Medical Inc.) is a body positioning device (Figure 1) that was introduced in November 2022 to increase patient stability during ERCP. Owing to the limited availability of the device, some procedures were conducted without it, resulting in a mixed sample. This device involves a beads‐filled mattress that conforms to the patient body, distributing pressure evenly. Pressing a dedicated compressor switch releases air from the mattress, causing it to harden through moderate decompression, thus maintaining the patient's body position. The compressor stops automatically during this process so that the patient does not experience excessive pressure. If necessary, the mattress can be reinflated during the procedure, and the compressor will automatically reactivate, maintaining the desired level of immobilization. After the procedure, turning off and disconnecting the compressor quickly allows the mattress to return to its softened state. The patient was initially positioned in the prone position. After adequate sedation was confirmed, diclofenac was administered rectally, and the procedure started. During the endoscope passage through the pharynx, the head was turned sideways with slight shoulder elevation. If the pyloric passage was difficult, a semi‐lateral position was used to facilitate advancement. Once the endoscope reached the papilla of Vater, the patient was repositioned in the prone position, and the Medo V‐Fix device was used for stabilization. Writing informed consent for the use of the device was obtained before the examination.

### ERCP procedure

ERCP was performed by trainees under the supervision of experienced endoscopists at Toyonaka Municipal Hospital, a Japan Gastroenterological Endoscopy Society‐certified teaching hospital. Cannulation was performed using a wire loading method with a side‐viewing duodenoscope (TJF‐Q290V; Olympus Optical Co. and ED‐580T; Fujifilm). Diclofenac was administered rectally to prevent post‐ERCP pancreatitis, and the dosage was adjusted according to body weight (25 or 50 mg), provided that there were no contraindications.

### Sedation and analgesia

Sedation was achieved with midazolam and pethidine hydrochloride. The initial dose of midazolam ranged from 2 mg to 3 mg, adjusted for patient weight and age. The initial dose of pethidine ranged from 7 to 10.5 mg, and an additional dose was provided as needed. Thiopental was used when midazolam or pethidine was insufficient for adequate sedation. These initial doses represented the scheduled doses for sedation, with additional doses administered as needed based on the patient's clinical response. However, adjustments made to maintain appropriate sedation over prolonged periods were not categorized as deviations from the scheduled dose. BIS monitoring was not utilized in this study. Sedation adequacy was assessed based on clinical judgment, including patient responses and respiratory status. Initial doses were administered with the goal of achieving moderate sedation (Ramsay Sedation Scale 4–5).

### Monitoring during ERCP

During ERCP, patients were monitored by at least one nurse, the endoscopist, and an assistant. If significant patient movement compromised monitoring, an additional nurse was called, or the procedure was discontinued. During the examination, the nurse monitored the patient's general condition, breathing, blood pressure, heart rate, and saturation of percutaneous oxygen (SpO_2_) and recorded the patient's progress and any SRCs.

### Body motion evaluation

Body movement control was categorized into three grades:

‐ Good: Patient movement was effectively controlled with the scheduled sedative doses.
‐ Poor: Patient movement was controlled with higher‐than‐scheduled sedative doses, excluding additional doses administered required to maintain appropriate sedation over a prolonged period.
‐ Very Poor: Patient movement was controlled with the assistance of additional personnel despite additional sedative doses.

### Outcomes

The primary outcome was the grade of body movement control during ERCP. Secondary outcomes included the total dose of sedatives administered, the rate of procedure discontinuation due to inadequate movement control; and the incidence of SRCs, such as hypoxemia, hypotension, and bradycardia. As data regarding SRCs were obtained retrospectively from nursing records, it was difficult to ascertain whether their documentation was objective or accurate. However, hypoxemia was diagnosed based on a decrease in SpO₂ from baseline, as determined by the endoscopist, and the need for oxygen administration. Hypotension was diagnosed on the basis of a decrease in blood pressure below 90 mmHg from baseline or the need for intravenous fluid administration. Bradycardia was diagnosed on the basis of a heart rate of less than 50 bpm. Additional outcomes included procedure duration and radiation exposure metrics such as fluoroscopy time and the air kerma dose‒area product.

### Statistical analysis

Continuous variables are expressed as medians and interquartile ranges (IQRs), and categorical variables are presented as counts and percentages. Sample size calculations were not performed because of the retrospective nature of the study and the lack of prior evidence on which to base them. The Wilcoxon test was used to compare continuous variables, and categorical variables were compared using the *χ* ^2^ test or Fisher's exact test, as appropriate. Trends in body movement control (good/poor/very poor) were assessed using the Cochran‒Armitage trend test. Bonferroni correction was applied to adjust for multiple comparisons in the three‐group comparison of outcomes (“Good,” “Poor,” and “Very Poor”), and a *p‐*value < 0.0167 was indicative of statistical significance.

Logistic regression analysis was conducted to identify predictive factors of good body movement control, with seven variables evaluated: sex, age 75 years or older, BMI of 25 or higher, device use, emergency setting, first papilla, benign disease or malignancies, and postoperative stomach status. Propensity scores for good body movement control were calculated using these significant factors in a multiple logistic analysis, and a 1:1 matched study group was created with a caliper width of 0.05 to minimize selection bias. The device and nondevice groups were compared. A *p*‐value <0.05 indicated statistical significance. Analyses were performed using JMP software (version 17.0.0; SAS Institute).

## RESULTS

### Patient characteristics and basic ERCP‐related parameters

The baseline characteristics of the patients are summarized in Table 1. The median age of the patients was 78 years (IQR: 71–84 years), and 59% were male. The median body weight was 56.0 kg (IQR: 47.7–63.0 kg). A total of 543 patients (35.5%) had naïve papillae. Diclofenac was administered to 1369 patients (51.2% received 50 mg, 41.8% received 25 mg), whereas 7.1% did not receive diclofenac. The main indications for ERCP included common bile duct stones (45.9%), pancreatic cancer (22.1%), and cholangiocarcinoma (13.3%). Scheduled procedures accounted for 77.5%, whereas 22.6% were emergency procedures. The median cannulation time was 2 min, and the median procedure time was 31 min (IQR: 20–49 min; Table 1).

**TABLE 1:** Patient characteristics.

Parameters	Total
Number of patients, *n*	1528
Use of the Medo V‐Fix, *n* (%)	697 (45.6)
Patient factors	
Age, years (IQR)	78 (71–84)
Sex, male, *n* (%)	904 (59.2)
Weight, kg (IQR)	56.0 (47.7–63.0)
BMI (IQR)	22.0 (19.6–24.1)
Diseases and malignancies, *n* (%)	844 (55.2)
Common bile duct stones, *n* (%)	701 (45.9)
Pancreatic cancer, *n* (%)	337 (22.1)
Cholangiocarcinoma, *n* (%)	203 (13.3)
Gallbladder cancer, *n* (%)	56 (3.7)
Other malignant diseases, *n* (%)	96 (6.3)
ERCP procedure factors	
Setting	
Emergency/scheduled, *n* (%)	345 (22.6)/1139 (77.5)
Naïve papilla, *n* (%)	543 (35.5)
Surgically altered anatomy, *n* (%)	96 (6.7)
Premedication, diclofenac	
None/25 mg/50 mg, *n* (%)^*^	104 (7.1)/615 (41.8)/754 (51.2)
Time to reach papilla, min (IQR)	3 (2–6)
Intubation time, min (IQR)	2 (1–9)
Treatment time, min (IQR)	26 (15–43)
Total procedure time, min (IQR)	31 (20–49)
Radiation related parameters	
Fluoroscopy time, min (IQR)	10 (6–17)
*K*_a,r_, mGy (IQR)	36.0 (20.9–62.5)
*P*_KA_, Gy cm^2^ (IQR)	7.4 (4.4–12.1)
No. of images per exam, median (IQR)	5 (3, 7)

Abbreviations: ERCP, endoscopic retrograde cholangiopancreatography; IQR, interquartile range.

* Missing data: *n* = 55.

### Comparison of outcomes with and without a body positioning device

Table 2 presents the characteristics of the ERCP‐related parameters between the device and nondevice groups. There were no significant differences between the two groups in terms of sex, weight, malignant disease prevalence, procedure setting, or the presence of naïve papilla, but the patients were significantly younger (78 vs. 77 years, *p* = 0.0210) and the cannulation time was longer (3 vs. 2 min) in the device group.

**TABLE 2:** Comparison of endoscopic retrograde cholangiopancreatography‐related parameters and body movement control between the device and nondevice groups.

	Use of the Medo V‐Fix	Without the Medo V‐Fix	*p‐*value
Number of patients	697	831	
Patient factors			
Age, years (IQR)	78 (71–85)	77 (70–83)	0.0216
Sex, male, *n* (%)	427 (61.3)	477 (57.4)	0.1261
Weight, kg (IQR)	55.9 (47.9–63.2)	56.2 (46.9–63.2)	0.4890
BMI (IQR)	22.1 (19.5–24.1)	22.1 (19.5–24.1)	0.9498
Diseases and malignancies, *n* (%)	312 (44.8)	380 (45.7)	0.7059
ERCP procedure factors			
Setting			
Emergency/scheduled, *n* (%)	98/355	194/603	0.2829
Naïve papilla, *n* (%)	122 (23.4)	253 (32.1)	0.4185
Cannulation time, min (IQR)	3 (1, 10)	2 (1, 7)	0.0184
Procedure time, min (IQR)	26 (16, 43)	27 (15, 42)	0.4850
Total procedure time 30 min and more, *n* (%)	368 (52.8)	441 (53.1)	0.7599
Radiation‐related procedures			
Fluoroscopy time, min (IQR)	10 (6, 17)	10 (6, 17)	0.9427
*K*_a,r_, mGy (IQR)	36.0 (21.5–61.6)	36.0 (20.2–64.7)	0.8998
*P*_KA_, Gy cm^2^ (IQR)	7.6 (4.6–12.6)	7.6 (4.4–12.7)	0.2347
Body motion evaluation			
Good/poor/very poor, *n* (%)	458 (65.7)/167 (24.0)/72 (10.3)	400 (48.1)/255 (30.7)/176 (21.2)	<0.0001

Abbreviations: ERCP, endoscopic retrograde cholangiopancreatography; IQR, interquartile range; K_a,r_, air kerma at the patient irradiation reference point; P_KA_, area air kerma integrated value.

### Body movement control

**FIGURE 2:**
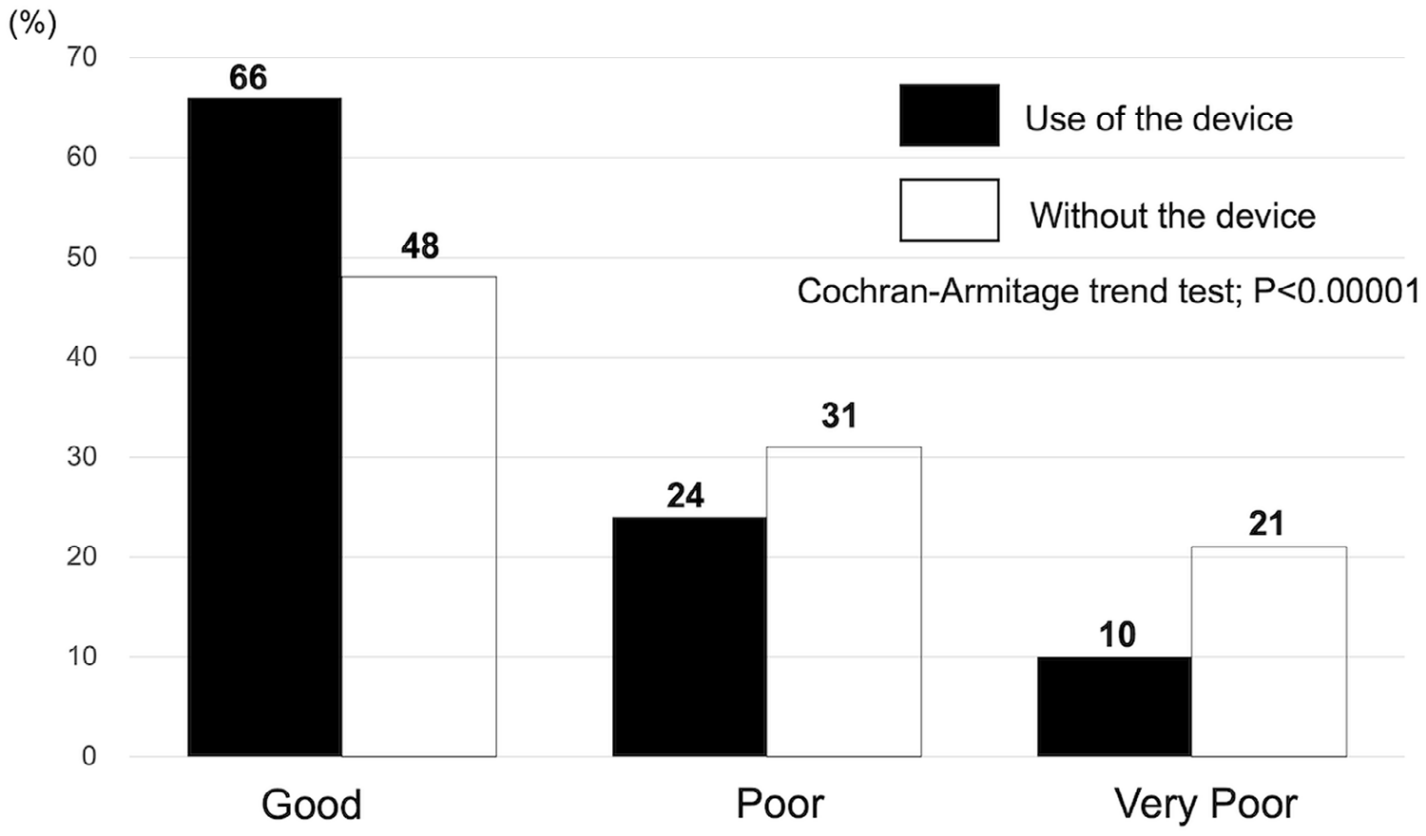
Trend analysis of body movement control in the device and nondevice groups. The proportion of patients with good body movement control was greater in the device group (65.7%) than in the nondevice group (48.1%), whereas very poor outcomes were less common with the device (10.3%) than without it (21.2%). The Cochran–Armitage trend test confirmed a significant improvement in body movement control with the device (*p* < 0.0001), indicating a favorable trend toward better procedural stability when using Medo V‐Fix.

The proportion of patients whose body movement was effectively controlled was significantly greater in the device group than in the nondevice group (good: 65.7% vs. 48.1%, *p* < 0.0001). Conversely, the proportions of patients whose body movement was poorly and very poorly controlled were significantly greater in the nondevice group than in the device group (poor: 30.7% vs. 24.0%, *p* = 0.0034; very poor: 21.2% vs. 10.30%, *p* < 0.0001; Table 3). The Cochran‒Armitage trend test confirmed a significant improvement in body movement control in the device group (*p* < 0.0001, Figure 2). This finding shows the effectiveness of the Medo V‐Fix device in achieving better outcomes for body movement control.

**TABLE 3:** Comparison of patient outcomes between the device and nondevice groups.

	*n*	Good^*^	Poor^†^	Very poor^¶^
Use of the Medo V‐Fix, *n* (%)	697	458 (65.7)	167 (24.0)	72 (10.3)
Without the Medo V‐Fix, *n* (%)	831	400 (48.1)	255 (30.7)	176 (21.2)

* *p* < 0.0001, “good” versus the others.

^†^ *p* = 0.0034, “poor” versus the others.

^¶^ *p* < 0.0001, “very poor” versus the others.

Logistic regression analysis was performed to identify factors associated with good body movement control. The univariate analysis revealed that the factors associated with better body movement control were age over 75 years (odds ratio [OR] 1.61, 95% confidence interval [CI]: 1.31–1.98, *p* < 0.0001), use of the device (OR 2.07, 95% CI: 1.68–2.54, *p* < 0.0001), emergency setting (OR 1.79, 95% CI: 1.39–2.30, *p* < 0.0001), and noninitial ERCP (OR 1.46, 95% CI: 1.18–1.81, *p* = 0.0005).

The multivariate analysis revealed that the significant predictors of good body movement control were female sex (OR 1.27, 95% CI: 1.01–1.60, *p* = 0.0400), age over 75 years (OR 1.27, 95% CI: 1.01–1.60, *p* = 0.0399), use of the device (OR 1.95, 95% CI: 1.56–2.44, *p* < 0.0001), emergency setting (OR 1.95, 95% CI: 1.47–2.58, *p* < 0.0001), and noninitial ERCP (OR 1.51, 95% CI: 1.20‐1.90, *p* = 0.0004). Device use and emergency setting had the highest odds ratios for predicting good body movement control (Table 4).

**TABLE 4:** Factor analysis to predict good body movement control.

		Univariate analysis		Multivariate analysis	
	Reference	Odds ratio (95% CI)	*p*‐value	Odds ratio (95% CI)	*p*‐value
Sex, female	Male	1.23 (1.00–1.51)	0.0510	1.27 (1.01–1.60)	0.0400
Age, 75 years and more	Less than 75 years	1.61 (1.31–1.98)	<0.0001	1.27 (1.01–1.60)	0.0399
BMI, 25 and more	Less than 25	1.28 (0.97–1.68)	0.0801	1.25 (0.93–1.67)	0.1363
Use of the Medo V‐Fix	Without the device	2.07 (1.68–2.54)	<0.0001	1.95 (1.56–2.44)	<0.0001
ERCP setting, urgent	Scheduled	1.79 (1.39–2.30)	<0.0001	1.95 (1.47–2.58)	<0.0001
Noninitial ERCP	Naïve papilla	1.46 (1.18–1.81)	0.0005	1.51 (1.20–1.90)	0.0004
Diseases, malignancies	Benign	1.08 (0.88–1.33)	0.4416	1.16 (0.93–1.46)	0.1915
Surgically altered anatomy	Normal	0.89 (0.59–1.34)	0.5726	1.00 (0.63–1.57)	0.9896

Abbreviation: ERCP, endoscopic retrograde cholangiopancreatography.

### Additional sedation requirements and procedure discontinuation‐ and SRCs

Significantly fewer patients required additional use of thiopental in the device group than in the nondevice group (9.5% vs. 15.6%; *p* = 0.0003). Additionally, the median dose of thiopental was lower in the device group (4.5 mg) than in the nondevice group (6 mg; *p* = 0.0015). There were no procedural discontinuations in the device group and five discontinuations in the nondevice group, although the difference was not significant (*p* = 0.0669; Table 5).

**TABLE 5:** Comparison of the use of sedative and analgesic drugs and the incidence of sedation‐related complications between the device and nondevice groups

	Use of the Medo V‐Fix, *n* = 697	Without the Medo V‐Fix, *n* = 831	*p*‐value
Use of sedative and analgesic drugs			
Dose of midazolam, mg (IQR)	4 (3, 5)	4 (3, 5)	0.1904
Dose of pethidine, mg (IQR)	35 (21, 35)	35 (24.5, 35)	0.2564
Use of thiopental, *n* (%)	66 (9.5)	130 (15.6)	0.0003
Dose of thiopental, mg (IQR)	4.5 (3, 8)	6 (4, 9.3)	0.0015
Any sedation‐related complications, *n* (%)	134 (19.2)	104 (12.5)	0.0003
Hypoxemia, *n* (%)	101 (14.5)	73 (8.8)	0.0005
Hypotension, *n* (%)	23 (3.3)	21 (2.5)	0.3683
Bradycardia, *n* (%)	13 (1.9)	10 (1.2)	0.2900
Tachycardia, *n* (%)	2 (0.3)	5 (0.6)	0.3655
Arrhythmia, *n* (%)	1 (0.1)	0 (0)	0.3773
Temporary cardiac arrest, *n* (%)	1 (0.1)	0 (0)	0.2747
Discontinuation due to body movement, *n* (%)	0 (0)	5 (0.6)	0.0669

Abbreviation: IQR, interquartile range.

### Sedation‐related complications and adverse events

During the study period, no mortalities or severe SRCs were reported. Additionally, no adverse events related to the body positioning device were observed. However, complications occurred significantly more often in the device group than in the nondevice group (19.2% vs. 12.5%, *p* = 0.0003). Among these SCRs, hypoxemia was significantly more common in the device group than in the nondevice group (14.5% vs. 8.8%, *p* = 0.0005; Table 4).

### Propensity score matching analysis

Multivariate logistic analysis revealed that device use, female sex, age over 75 years, and noninitial ERCP were significant predictors of good body movement control. To further assess the impact of the device, a 1:1 propensity score matching analysis was performed, resulting in 1246 matched patients. Table 6 shows the results of the propensity score matching analysis. After matching for significant predictors other than the device use, there were no significant differences between the two groups in terms of baseline characteristics. However, even after matching, the incidence of SRCs, including hypoxemia, was significantly higher and body movement control was significantly better in the device group (Table 6).

**TABLE 6:** Comparison of endoscopic retrograde cholangiopancreatography‐related parameters and body movement control between the device and nondevice groups after propensity score matching.

	Matched pair		
	Use of the Medo V	Without the Medo V	*p*‐v1alue
Number of patients	623	623	
Patient factors			
Age, years (IQR)	78 (72–85)	78 (72–84)	0.6455
Sex, male, *n* (%)	377 (60.5)	379 (60.8)	0.9538
Use of sedative and analgesic drugs			
Dose of midazolam, mg (IQR)	4 (3–5)	4 (3–5)	0.3895
Dose of pethidine, mg (IQR)	35 (21–35)	10 (24.5–35)	0.3508
Use of thiopental, *n* (%)	63 (10.1)	87 (14.0)	0.0450
Dose of thiopental, mg (IQR)	5 (3, 8)	6 (4, 9)	0.0132
ERCP procedure factors			
Setting, Emergency, *n* (%)	133 (21.4)	135 (21.7)	0.9450
Naïve papilla, *n* (%)	233 (37.4)	229 (36.8)	0.8603
Procedure time, min (IQR)	26 (16–43)	27 (15–41)	0.3650
Body movement control			
Good/Poor/Very poor, *n* (%)	404 (64.9)/154 (24.7)/65 (10.4)	306 (49.1)/198 (31.8)/119 (19.1)	<0.0001
Any sedation‐related complications, *n* (%)	119 (19.1)	78 (12.5)	0.0018
Hypoxemia	88 (14.1)	56 (9.0)	0.0029

Abbreviations: ERCP, endoscopic retrograde cholangiopancreatography; IQR, interquartile range.

### Procedure and radiation metrics

No significant differences were observed between the two groups in terms of procedure time, fluoroscopy time, or air kerma dose‒area product (Table 2).

## DISCUSSION

The primary finding of this study was that the Medo V‐Fix device significantly improved body movement control during ERCP, increasing procedural safety and efficacy. Good control was achieved for 65.7% in the device group compared with 48.1% in the nondevice group. The device reduced the need for manual assistance and additional sedatives, as evidenced by fewer patients in the device group requiring thiopental and the lower median dose of thiopental. These findings remained significant after propensity score matching. The results suggest that integrating body‐positioning devices into ERCP procedures can significantly improve patient outcomes.

Our findings are consistent with those of Lee et al., who demonstrated that the use of a patient‐positioning device during ERCP reduced the required doses of propofol, shortened recovery times, and increased satisfaction among medical staff.^5^ Similarly, the Medo V‐Fix device not only improved body movement control but also decreased the need for additional sedatives, underscoring that body positioning devices can play a critical role in enhancing the safety and efficiency of ERCP procedures.

Adequate sedation and analgesia are crucial for safe ERCP, especially given the prone position required for the procedure.^6^, ^7^ While benzodiazepines and opioids are commonly used, there is no consensus on the optimal choice of sedatives due to variations in practice environments and available personnel.^2^, ^3^ Although the use of propofol is recommended by physicians who can manage the airway,^8^ it was not used in this study because of the risk of hypotension and the need for additional anesthesia support.

Our findings did not support the notion that a body positioning device hinders endoscopic manipulation or causes patient discomfort. No device‐related adverse events occurred, and no patients refused repeated use or reported stress from repeated use. However, SRCs, particularly mild hypoxemia, were observed more frequently in the device group but were effectively managed under routine monitoring, without requiring procedure discontinuation or additional interventions. All hypoxemic events were appropriately managed with standard oxygen supplementation and routine monitoring. Although discomfort and pain were not quantitatively assessed in this retrospective study, no patients explicitly reported such issues.

One possible explanation for the increase in hypoxemia is that the device may have restricted chest and abdominal movements, affecting respiratory motion and causing transient oxygen desaturation. Furthermore, the inclusion of mild cases in our definition of hypoxemia may have contributed to the observed higher incidence. Despite these findings, the absence of severe adverse events suggests that the observed hypoxemia did not compromise patient safety and remained within the bounds of manageable procedural risks. These results underscore the importance of continuous monitoring during ERCP to ensure early detection and prompt response to any transient hypoxemic events. Future prospective studies are warranted to explore the mechanisms by which body positioning devices may influence respiratory function during ERCP.

In this study, the incidence of SRCs was extracted from nursing records, so the exact timing of hypoxemia is unclear. Furthermore, some cases of hypoxemia were observed even before the initiation of the body positioning device, highlighting the need for prospective studies. Nevertheless, since no severe SRCs were observed in the device group, this study demonstrated that the benefits of using the device for improved body movement control outweigh the risks. In the future, research intended to ascertain whether the use of body positioning devices, particularly in elderly patients, can reduce the amount of sedatives needed for adequate sedation is needed.

Patients and surgical staff are exposed to substantial doses of radiation during ERCP^9^, ^10^; however, it is worth mentioning that radiation doses vary across institutions owing to differences in procedural complexity and advanced equipment used.^11^, ^12^ Although no significant differences in fluoroscopy metrics were observed between the groups, the Medo V‐Fix device reduced the incidence of very poor body movement control, and very poor body movement control often requires manual stabilization near the radiation tube (10.3% vs. 21.2%, *p* < 0.001). Consequently, staff may be exposed to lower doses of radiation, thus reducing risks associated with radiation exposure.

Factors predicting good body movement control were analyzed using multivariate analysis and included female sex, device use, emergency setting, and noninitial ERCP. The results demonstrated that device use was significantly associated with improved body movement control, even after adjusting for pretest factors. These findings suggest that the benefits of using a body positioning device may extend beyond simple physical immobilization. Additionally, emergency settings and noninitial ERCP may reflect procedural efficiency or other time‐related factors, contributing to better control.

Despite its advantages, the Medo V‐Fix device has limitations. It may be less effective in patients with severe kyphosis or obesity because of suboptimal body contouring. Additionally, in cases of hypoxemia, the firm immobilization of the torso by the device may obscure visual assessment of respiratory effort, necessitating alternative monitoring strategies. Furthermore, inadequate sedation may require additional manual head stabilization, potentially disrupting the procedural workflow. Finally, repeated disinfection and frequent use can impact the durability, which increases costs.

This study has several limitations. First, as a single‐center retrospective study, the findings may not be generalizable to other settings. Additionally, detailed documentation of interventions, such as device deactivation or positional changes in response to hypoxemia, as well as patient discomfort or pain, was lacking. However, the inclusion of over 500 procedures involving the use of the Medo V‐Fix device strengthens the validity of the results. Second, the evaluation of body movement control was subjective and varied among examiners, which may have affected the accuracy of the comparisons.

## CONCLUSION

The Medo V‐Fix body positioning device improves body movement control and reduces the need for sedatives, increasing procedural safety and efficiency. The device reduced the incidence of poor body movement control by half, thereby reducing manual intervention. Although mild hypoxemia was more frequent, it was manageable with routine monitoring. These findings support integrating devices into standard ERCP practices to improve outcomes.

## CONFLICT OF INTEREST STATEMENT

None.

## ETHICS STATEMENT

This study was approved by the Ethics Committee of the Toyonaka Municipal Hospital (2024‐05‐02). As this was a retrospective study that used previously collected personal data, the need for informed consent was waived through the opt‐out method on our hospital's website.
